# Cardiac Progenitor Cells in Basic Biology and Regenerative Medicine

**DOI:** 10.1155/2018/8283648

**Published:** 2018-02-05

**Authors:** Nevin Witman, Makoto Sahara

**Affiliations:** ^1^Department of Cell and Molecular Biology, Karolinska Institutet, 171 77 Stockholm, Sweden; ^2^Department of Medicine-Cardiology, Karolinska Institutet, 171 77 Stockholm, Sweden

## Abstract

Major cardiovascular events including myocardial infarction (MI) continue to dominate morbidity rates in the developed world. Although multiple device therapies and various pharmacological agents have been shown to improve patient care and reduce mortality rates, clinicians and researchers alike still lack a true panacea to regenerate damaged cardiac tissue. Over the previous two to three decades, cardiovascular stem cell therapies have held great promise. Several stem cell-based approaches have now been shown to improve ventricular function and are documented in preclinical animal models as well as phase I and phase II clinical trials. More recently, the cardiac progenitor cell has begun to gain momentum as an ideal candidate for stem cell therapy in heart disease. Here, we will highlight the most recent advances in cardiac stem/progenitor cell biology in regard to both the basics and applied settings.

## 1. Introduction

Due to marginal improvements in heart failure treatments, a greater number of elderly patients are living longer with chronic heart failure. However, no treatment regime is capable of fully reversing pathological remodeling or completely restoring ventricular function after a major cardiovascular event, such as MI. In fact, many patients progress steadily towards New York Heart Association (NYHA) class III-IV heart failure where the only curative therapy is heart transplantation. Due to the unbalanced need of donor hearts, alternative regenerative therapeutic approaches aim to build up lost functional ventricular muscle.

Cell-based therapies have been conceptualized to alleviate some of the barriers limiting cardiac regeneration. The golden objective in cell-based therapies is to repopulate parts of damaged myocardium with engrafted, functional cells that restore lost cardiac function, enabling sufficient oxygen and nutrient circulation to all the vital organs of the body. Several technological, financial, and ethical hurdles impede such a medicinal feat, yet the field continues to move forward with the collaborative efforts between stem cell biologists, who are investigating novel mechanisms of cardiac regeneration, and medical teams in cardiology.

**Figure 1:**
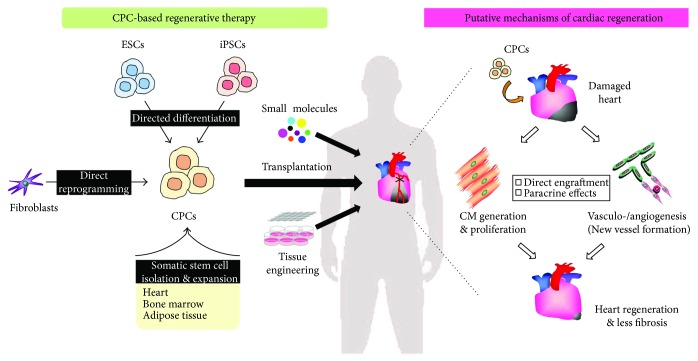
CPC-based regenerative therapy for heart disease. Cardiac progenitor cells (CPCs) can be obtained through several approaches (left). Directed differentiation of pluripotent stem cells such as embryonic stem cells (ESCs) or induced pluripotent stem cells (iPSCs) can generate “developmental (embryonic)” CPCs, while isolation and expansion of tissue- (i.e., heart) resident stem/progenitor cells can generate “adult” CPCs. Recently, an alternative approach by employing direct reprogramming can also generate “inducible” CPCs. These purified and expanded CPCs combined with small molecules and/or tissue engineering can be therapeutically transplanted into the damaged hearts of patients, such as those suffering from ischemic cardiomyopathy. Putative cellular mechanisms of cardiac regeneration by CPC-based therapy (right). Transplanted CPCs can be engrafted directly into the damaged host cardiac tissue and differentiated into mature cardiomyocytes as well as vascular cells (smooth muscle cells and endothelial cells). Simultaneously, the CPCs can potentially promote proliferation of preexisting cardiomyocytes in the damaged heart and also induce vasculo-/angiogenesis in the ischemic regions through secretion of the paracrine factors. Theoretically, increased working cardiomyocytes and newly formed vessels could lead to effective heart regeneration and a reduction in cardiac fibrosis in a coordinated fashion. Further details for cell-free approaches (e.g., small molecules and tissue engineering), somatic stem cell-expansion derived from bone marrow and adipose tissue, and CPC therapy-related mechanisms for cardiac regeneration have been reviewed elsewhere [6, 7].

Much effort has been made in replacing damaged myocardium with adult/mature cardiomyocytes (CMs), those of which are derived from pluripotent stem cells or reprogramming strategies [1, 2]. However, several major technical limitations are compromising the success of an implantable, mature, cardiac muscle patch, including low numbers of surviving implanted CMs and the lack of electromechanical and structural integration between the host and donor CMs [3, 4]. More recently, emerging scientific evidence has begun to emphasize the use of cardiac progenitor cells (CPCs), rather than differentiated CMs, as a novel treatment strategy for cardiac regeneration. This is due to the notion that CPCs, which imply both embryonic/developmental and adult CPCs, are more capable of engrafting to host myocardium, in part by their strong proliferative potential and also their ability to generate multiple cardiac derivatives (Figure 1). Unlocking the use of such CPC technologies could potentially eliminate the limitations seen with mature CMs and provide long-term therapeutic effects, although the CPC therapy may bring the new challenges of obtaining efficient and committed differentiation of CPCs into CMs *in vivo* under pathological conditions, such as the ischemic and/or injured microenvironment [3, 5].

In this minireview, we discuss briefly the recent advances and knowledge of CPCs in basic biology and also clinical settings. For a more in-depth review of cell-free and cell-based approaches to cardiac regeneration, we refer the reader to the following reviews [6, 7].

## 2. Embryonic and Adult Cardiac Progenitor Cells

Conceptually, there are two distinct types of CPCs: embryonic/developmental CPCs and adult CPCs [8, 9]. Embryonic CPCs exist in the developmental mammalian heart, where they derive from a common mesodermal lineage. During cardiac development, two heart fields emerge termed the First Heart Field (FHF) and Second Heart Field (SHF). The FHF forms the cardiac crescent at embryonic day (E) 7.5 in mice and during embryonic days 16 to 18 in human and is marked by the transcription factor *NKX2-5* [9, 10] and the cyclic nucleotide-gated ion channel *HCN4* [11, 12]. The FHF then fuses at the midline and eventually forms the primitive heart tube that will begin to pump blood. The SHF is instead specifically marked by Islet-1 (*ISL1)* expression and lies medially and posteriorly to the crescent/FHF [13]. The SHF progenitors migrate behind the heart tube and extend anteriorly and posteriorly into the pharyngeal mesoderm to lengthen the outflow tract and form the looping heart tube at E8.5–9.0 in mice and during embryonic days 23 to 28 in human, in concert with the FHF progenitors [8, 14, 15]. FHF derivatives give rise to left ventricular myocardium with partial contribution to the atria, whereas SHF derivatives contribute to myocardium of the right ventricle, parts of the atria, and the outflow tract. The CPCs derived from the FHF and SHF will go on to give rise to many of the intermediates that are responsible for generating all the major cell types in the heart, including CMs, vascular smooth muscle cells (SMCs), arterial and venous endothelial cells (ECs), fibroblasts, and conductive cells of the cardiac conduction system. Much work is currently ongoing to understand the molecular underpinnings that regulate the spatiotemporal aspects of multipotent CPCs, as well as the signals that promote their differentiation into the diverse cell types that create the beating heart [16].

In addition to embryonic FHF and SHF CPCs, other progenitor cell populations, including epicardium-derived cells (EPDCs) and cardiac neural crest cells (cNCCs), also contribute to the formation of the developmental heart. Embryonic EPDCs are likely to contribute the SMCs, ECs, fibroblasts, and a small population of CMs in the heart through epithelial-to-mesenchymal transition, although EPDCs are heterogeneous and their contribution to CMs is still under debate [17–19]. cNCCs, which originate from the dorsal neural tube and migrate through the posterior pharyngeal arches to the arterial pole of the heart tube, give rise to SMCs of the outflow tract and contribute to outflow tract septation and valve formation [20, 21].

Embryonic-like CPCs, which are referred to as “developmental” CPCs, can be generated *in vitro* from pluripotent stem cells such as embryonic stem cells (ESCs) or induced pluripotent stem cells (iPSCs) [3, 5, 6]. CPCs in general are defined by having self-renewing and clonogenic properties, as well as multipotent differentiation capabilities to give rise to different cardiac lineages such as CMs, SMCs, and ECs, both *in vitro* and *in vivo* [22].

In contrast to the embryonic/developmental CPCs, to date, several kinds of endogenous CPCs, referred to as “adult” CPCs, have been isolated from adult rodent and human hearts, although their role in homeostasis or potential reparative function remains controversial [23]. The cell-surface marker tyrosine kinase receptor *c-kit* has been routinely used to identify the adult CPCs [22, 24]. Cardiac c-kit^+^ cells isolated from adult human heart and injected into the infarcted rodent myocardium have been shown to increase cardiac function and improve cardiac structure [24, 25]. However, more recently, it was reported that very few cardiomyocytes are generated from c-kit^+^ cells based on genetic lineage tracing technology [26], although in the mouse model used in [26], all the c-kit^+^ cells were constitutively tagged, and thereby, the cardiac-derived c-kit^+^ cells localized in the ageing or injured heart could not be distinguished from the bone marrow-derived c-kit^+^ cells identified in the heart. There continues to be abundant controversy around the origin of c-kit^+^ cells as they are broadly expressed in cells of the hematopoietic lineage [27], and a large number of c-kit^+^ cells in the heart after MI appear to be bone marrow-derived [28]. Interestingly, the latest report has revealed that majority (≈90%) of the resident c-kit^+^ cells in the rodent heart are blood/endothelial lineage-committed cells, while cardiac c-kit^+^ (blood/endothelial lineage-negative) cells represent ≤ 10% of the total c-kit^+^ cells in the heart [29]. It is speculated that the positive effects seen from the delivered c-kit^+^ cells in the post-MI setting could be due to the release of signaling molecules, rather than the engrafted cells themselves [30, 31].

Previously, seminal works identified vascular endothelial growth factor type 2 receptor Flk-1, also known as kinase insert domain protein receptor (KDR) in human, and the platelet-derived growth factor receptor alpha (PDGFR-*α*) as some of the earliest cardiovascular progenitor cell markers involved in early stages of human cardiac development [32–35]. To date, a KDR^+^/PDGFR*α*^+^ population has become widely accepted as a classical CPC marker profile. Researchers are using this population and others as a means to enrich cardiac progenitors to possibly enhance applications of downstream cell-based therapies and disease modeling.

Apart from c-kit^+^ or KDR^+^/PDGFR*α*^+^ cells, additional progenitor-like cell populations have been identified as adult CPC-like cells, including Sca1^+^ cardiac cells [36, 37], cardiosphere-derived cells [38], and cardiac side population cells [39]. These cell types are heterogeneous in nature, and populations identified with different markers or approaches may have both unique and overlapping subsets in regard to molecular and physiological characteristics.

## 3. Recent Findings of Embryonic CPCs

A multipotent progenitor cell type that can intrinsically expand within the cardiac lineage has great potential as a regenerative therapy. In order to employ the correct cell type for regenerative purposes against heart disease, it is imperative to understand the role of the CPCs in development. Embryonic/developmental CPCs can be found in early embryonic stages of cardiac development, as mentioned above; however, they can also be generated *in vitro* from pluripotent stem cell technologies. The assessment of such CPCs both *in vivo* and *in vitro* provide a means for answering unresolved questions about the diversity and commitment of their nature. Furthermore, advanced technologies involving elegant lineage tracing strategies, deep RNA-sequencing tools, and CRISPR-CAS genome editing have allowed researchers to better identify new and novel markers of the embryonic CPCs [40]. Below, we will highlight several recent papers that have elucidated novel markers and molecular mechanisms of embryonic CPCs through a combination of these technologies.

A report by Jain et al. identified a transcription factor Hopx^+^ cell population that is committed to cardiomyocyte fate [41]. By employing a knock-in approach, the authors showed that *Hopx* expression initiates shortly after the expression of FHF marker *Nkx2-5*. The use of fate-mapping experiments illustrated that Hopx^+^ cells were distributed in all four chambers of the developing heart, and the Hopx^+^ derivatives were comprised entirely of cardiac myocytes. The mechanism by which Hopx promotes myogenesis through the repression of Wnt signaling was clearly elucidated by employment of a previously published *in vitro* ESC differentiation protocol [34]. Finally, the authors showed that Hopx deficiency gave rise to a thinning myocardium and cardiac rupture in developing mouse embryos. Whether genetic alterations in the *Hopx* gene could give rise to similar congenital impairments during human cardiac development remains elusive, yet the discovery of a specific CPC subtype that gives rise solely to cardiac muscle could provide profound insights for rebuilding damaged and/or atrophic myocardium.

More recently, another report highlighted a member of the forkhead class of DNA-binding proteins, Foxa2, as a marker of a novel progenitor population, which unlike that of the 4-chamber cardiac identifier Hopx gave rise primarily to CMs exclusively in the ventricles [42]. The use of a Foxa2 lineage tracing model system clearly revealed the expression profile of Foxa2, which was found predominantly in the node, midline, and visceral endoderm as well as regions of migrating mesoderm cells during late stages of gastrulation at E7.5 in mice. As the heart continued to develop into four distinct chambers (E9.5–E17), Foxa2^+^ derivatives became localized to the ventricular chambers, with very few being expressed in the atria. Next, Bardot et al. [42] employed a murine ESC cardiac differentiation protocol in order to see if embryonic Foxa2^+^ CPCs could be generated *in vitro*. The group showed that a large portion of a KDR^+^/PDGFR*α*^+^ CPC population also coexpressed Foxa2. By employing cardiovascular lineage analysis together with immunohistochemistry and flow cytometry, Foxa2 expression was revealed predominantly in the ventricular CMs and in equal proportions between the left and right ventricles [42].

Work produced by Ishida et al. showcased that Gfra2 (GPI-anchored neurotrophic factor receptor) expression labels a specific population of embryonic CPCs in mouse cardiac development, which is required for cardiac compaction [43]. According to single-cell profiling studies during murine heart development, the authors showed that Gfra2 was coexpressed with Mesp1, a well-known early cardiac mesodermal marker. Using whole mount in situ hybridization studies and immunohistochemistry procedures, Ishida et al. showed the localization of Gfra2 and concluded a Gfra2 expression pattern that labels some subsets of embryonic CPCs in both the FHF and SHF. The authors also demonstrated that Gfra2 expression marks a human developmental CPC population during ESC/iPSC differentiation. The expression profile of the *Gfra2* gene appears to peak just before embryoid bodies begin to beat in culture. The proportion of KDR^+^/PDGFR*α*^+^ cells expressing Gfra2 is quite low but give rise to mature CMs. However, in their differentiation protocol, a Gfra2-negative KDR^+^/PDGFRα^+^ population failed to give rise to differentiated CMs, supporting the notion of a strong specificity of Gfra2 to give rise to a distinct CPC population [43]. Furthermore, the emergence of a surface receptor to label and isolate embryonic (or adult) CPCs is enticing for future cell-based therapies, as many well-known markers of embryonic CPCs are transcription factors that require fixation of the cells for successful labeling and as such cannot be used for downstream *in vivo* applications.

## 4. Expansion, Maintenance, and Preclinical Use of Embryonic or Inducible CPCs

The creation of a technology platform capable of expanding a multipotent and clonogenic CPC population that produces mature cardiomyocytes and vascular cells has been challenging. The exploited accomplishment of which has direct implications in understanding developmental cardiogenesis, cardiac disease modeling, and regeneration research, as well as cardiotoxicity studies for novel pharmacological agents. Several recent reports have paved great progress in the field, and below, we will highlight a few selected works, showing novel findings for effectively expanding embryonic (developmental) or inducible CPCs and improving renewable cardiac precursor technologies.

A finding produced from the Mummery lab attractively illustrated a technique by which developmental CPCs could be restrained from further differentiation through the control of oncogene *Myc* expression and simultaneously expanded using IGF-1 and a hedgehog pathway agonist [44]. Using a human ESC line and a Tet-On system, the group could regulate expression of Myc in a fine-tuned manner with doxycycline administration during differentiation, thereby halting CM differentiation, whereas in the absence of doxycycline, the cells formed beating CMs. Birket et al. [44] also demonstrated long-term expansion of the developmental CPCs, undergoing over 40 population doublings, which did not alter the multipotent capacity of the CPCs; as even the highly expanded CPCs could generate large numbers of successfully differentiated CMs and ECs.

Yet more recently, two independent research groups reported two different strategies for the expansion of “inducible” CPCs from reprogrammed adult mouse fibroblasts [45–47]. Using a combination of transcription factors, which were 5 cardiac genes for direct reprogramming of fibroblasts into CPCs [45] or 4 Yamanaka factors for generating iPSC-like cells first, followed by committed differentiation into CPCs [47], and a defined media containing growth factors and small molecules, both groups were able to produce and maintain a cell population that was highly expandable and could give rise to CMs, ECs, and SMCs. The CPCs produced by both groups, referred to as “inducible” CPCs, could be expanded > 10^10^-fold under chemically defined conditions with BIO and LIF to activate the Wnt and JAK/STAT pathways, respectively [45], or with a JAK inhibitor and BACS (BMP4, Activin A, CHIR99021 (a GSK inhibitor), and SU5402 (an inhibitor of FGF, VEGF, and PDGF)) [47], allowing for the propagation and expansion of desirable cell numbers for *in vivo* experiments. Both Lalit et al. [45] and Zhang et al. [47] went on to demonstrate that morphologically, the delivery of the inducible CPCs can reduce major architectural remodeling and improve cardiac function when delivered to the murine heart at the onset of MI, which was depicted by decreased scar sizes several months following the injury and implantation. In the results, the inducible CPC-derived exogenous CMs were found engrafted deeply within the heart scar tissue where they exhibited expression of marker genes indicative to differentiated and mature CMs, and thereby, both groups concluded that the beneficial effects seen in these studies appear to be based on direct engraftment of the injected inducible CPCs *in vivo* [45, 47]. Further studies are needed to more clearly decipher the ideal transplantable number of the inducible CPCs, which can promote cardiac repair and enhance long-term engraftment *in vivo*.

A study from the Murry lab sought to directly compare the regenerative capabilities of implanted human cardiac cell types; cardiomyocytes derived from human ESCs (hESC-CM), cardiovascular progenitors derived from human ESCs and expressing KDR^+^/PDGFR-*α*^+^ (hESC-CVP), and human bone marrow mononuclear cells (BMMC). The group administered these cell populations at the onset of a reperfusion MI injury in the nude rat heart [48]. The study concluded that the administration of both hESC-CMs and hESC-CVPs were capable of improving cardiac function one month following the ischemic reperfusion injury, more efficiently than the human BMMCs. Interestingly, the hESC-CVPs did not appear to yield a larger graft or give rise to a more significant number of human vessels in the grafted region, compared with hESC-CMs. However, there may exist several issues regarding an ideal number of the transplanted cells as well as a special time window in which the developmental CPCs must be administered as to not lose their proliferative and regenerative properties, which the authors did not address. Further experiments with variations in cell numbers, different cell populations, and timings of administration are needed to reach a more valid conclusion.

## 5. Adult and Developmental CPCs in Clinical Trials

There has now been a multitude of clinical trials that have employed stem cell technologies for patients with ischemic cardiomyopathy, the findings of which support the use of stem cell therapies in the heart to be safe [49]. Infusions of bone marrow-derived cells (BMCs) represent the largest number of clinical studies for MI. There are many cell populations that fall under the BMC umbrella including hematopoietic stem cells (HSCs) and mesenchymal stem cells (MSCs). To report the findings of BMCs in clinical cardiac studies would outweigh the scope of this review; however, for a comprehensive overview of such clinical studies, we direct the reader to the following review [50]. Here, we will focus on the clinical trials using purified adult or developmental CPCs as a regenerative therapy for ischemic heart disease (Table 1).

The SCIPIO study was the first CPC clinical trial to investigate the therapeutic effects of autologous CPCs (cardiac c-kit^+^ cells) in patients with ischemic cardiomyopathy [51]. The cells were isolated from cardiac tissue of patients during surgery and expanded ex vivo, and later delivered via intracoronary infusion. Results from the SCIPIO trial showed an increase in several functional parameters and no evidence of tumor formation at 1 yr follow-up, although it must be noted that concerns regarding patient randomization and the integrity of certain data generated in the SCIPIO trial have been raised [52]. Following the SCIPIO trial, a new trial CONCERT-HF (NCT02501811) will aim to deliver a combination therapy utilizing both MSCs and cardiac c-kit^+^ cells for the treatment of ischemic cardiomyopathy, as MSCs have been shown to increase several parameters of cardiac function when administered to the heart after MI, effects of which are thought to be paracrine-mediated [53, 54].

Adult CPC-like cells can also be obtained through human myocardial biopsies, where cultured pieces of myocardial tissue give rise to spherical clusters of stem cell-like cells coined cardiospheres [38, 55]. Several phase I clinical trials including CADUCEUS and ALCADIA (NCT00981006) tested the efficacy and safety of intracoronary delivery of cardiosphere-derived cells (CDCs) in patients with ischemic cardiomyopathy and reported small improvements in regional but not global function, as well as decreased scar sizes [56, 57]. Although some concerns exist in regard to capillary plugging due to the size of the cardiospheres, several ongoing clinical trials including ALLSTAR (NCT01458405), HOPE (NCT02485938), and DYNAMIC (NCT02293603) are aiming to address the real regenerative potential of CDCs for ischemic cardiomyopathy and also to evaluate safe dosage limits as well as differences between an allogeneic and autologous cell source of CDCs.

An additional clinical trial CAREMI (NCT02439398) is currently ongoing to test the feasibility and safety of delivering an allogeneic adult CPC population in human, isolated from right atrial appendages and expanded *in vitro*.

Overcoming the technological hurdle of deriving functional CMs and their progenitors from ESCs/iPSCs is beginning to pave great insight for their potential uses in the clinic [58, 59]. Although most of human ESC and iPSC-derived CM protocols can give rise to efficient numbers of beating cells, much optimization is required to generate highly enriched populations of CMs devoid of alternate cell types or undifferentiated stem cells, at low cost and in a timely manner. It should be also noted that the difficulties of obtaining fully differentiated CMs from ESCs/iPSCs are frequently observed, as the previous report indicated immaturity of ESC/iPSC-derived CMs compared with native ventricular tissue-derived CMs [60]. Even with such drawbacks, the clinical trial ESCORT (NCT02057900) is recruiting patients with severe ischemic heart failure (LVEF ≤ 35%) in order to evaluate the regenerative effects of a human ESC-derived developmental CPC denoted by CD15^+^/ISL1^+^ coexpression. Patients will receive a fibrin gel embedded with the human ESC-derived CD15^+^/ISL1^+^ CPCs at the onset of coronary artery bypass grafting. The generation and survival of the patch, as well as the efficacy on patient cardiac function, will assess the overall feasibility of the study (Table 1).

## 6. Unresolved Issues and Future Perspectives

Overall, the use of CPCs as a regenerative therapy in the clinic to date has shown varying degrees of benefits; the outcomes of which we hope may one day provide alternate options when conventional medical treatments fail. Several engaging and ongoing clinical trials are still deciphering optimal cell types and doses, and we anxiously await the feasibility and safety of such approaches. However, before directly applying CPC therapy in the clinic, many critical issues, including the challenges of electrical coupling, undetermined mechanistic aspects, long-term engraftment, and the direct reprogramming of the (inducible) CPCs as an alternative approach, should all be addressed [6, 7].

One major caveat associated with the CPC/CM-based therapy is the risk of arrhythmias due to incomplete electrical coupling of the transplanted cells with the host cardiac tissue. Indeed, few studies have thoroughly evaluated the electrical integrity of the cardiac system following the administration of human ESC-derived CMs in ischemic models of nonhuman primates or guinea pigs, but those studies have obtained varying results [61–63]. Ideally, transplanted cells have to align, engraft, and couple with host cardiomyocytes in an ordered fashion. Further studies are required to determine how this process is precisely orchestrated [4].

The mechanisms of action by which CPCs contribute to the generation of new CMs, promotion of preexisting CM proliferation, and/or development of vasculo-/angiogenesis remain to be fully elucidated (Figure 1). It is commonly speculated that direct engraftment of the injected adult CPCs is a relatively rare event and that the functional benefits associated with the administration of the CPCs are derived predominantly from their paracrine effects [64, 65]. However, the latest studies have revealed that the transplanted inducible CPCs exert beneficial effects based on direct engraftment *in vivo*, as described above [45, 47]. To improve the long-term cell engraftment in the ischemic environment, cardiac tissue engineering with natural or synthetic biomaterials is most likely to serve as an excellent tool [66, 67]. Yet the potential paracrine effects, such as cytokines and growth factors released by the transplanted adult CPCs or human ESC-derived CMs, are still considered to be indispensable on the CPC/CM therapy-mediated cardiac protection and repair after injury [64, 65, 68].

Alternative approaches to cell therapies for cardiac repair also include reprogramming strategies using fibroblasts [2, 6]. Cardiac reprogramming of fibroblasts can be achieved through direct conversion by employing a unique combination of cardiac-specific transcription factors, miRNAs, and/or chemical molecules *in vitro* and *in vivo* [2, 6, 69, 70]. To date, these *in vivo* studies have shown only direct reprogramming of cardiac fibroblasts into an “induced CM-like cell” but not adequate CPCs, although several *in vitro* studies have shown direct reprogramming of fibroblasts into an “inducible CPC” (Figure 1) [45, 47]. Several reprogramming strategies to generate cardiac cell lineages from fibroblasts, including inducible CPCs to differentiated CMs *in vitro* and *in vivo*, continue to be investigated [71].

Regardless of the several critical issues as described above, the concept of enhancing stem cell properties through a combination of strategies could go some way in obtaining better outcomes for patients. An innovative focus that aims to synergize cell-based and cell-free therapies such as combining “ideal” CPC types with gene therapy, small molecules, and/or tissue engineering strategies should be conceptualized as a plausible clinical treatment for the enhancement of regenerative therapies in cardiovascular disease (Figure 1). Continuous and collective efforts by stem cell biologists and medical teams in cardiology must open the door and generate novel paths toward a goal of successfully establishing cardiac regenerative therapeutics in the near future.

## Figures and Tables

**Table 1:** Selected clinical trials employing CPC therapy for cardiac regeneration against ischemic cardiomyopathy.

Trial name/reference	Classification	Cell type	Delivery route	Patient number	Follow-up time	Outcome	Side effects
SCIPIO (Chugh et al., 2012)	Phase I	c-kit^+^ CPCs	Intracoronary	33	4 & 12 mo	LVEF: 8% ↑ at 12 mo versus baseline	None
						Scar size: 30% ↓ at 12 mo versus baseline	
CONCERT-HF (NCT02501811)	Phase II	c-kit^+^ CPCs & MSCs	Transendocardial	Est 144	6 & 12 mo	Currently ongoing	N/A
CADUCEUS (Malilarus et al., 2014)	Phase I	CDCs	Intracoronary	25	6 & 12 mo	LVEF: unchanged at 12 mo versus baseline	1 patient death
						Scar size: 12.3% ↓ at 12 mo versus baseline	
ALCADIA (NCT00981006)	Phase I	CDCs	Intramyocardial with CABG	6	12 mo	LVEF: 12% ↑ at 6 mo versus baseline	None
						Scar size: 3.3% ↓ at 6 mo versus baseline	
ALLSTAR (NCT01458405)	Phase I/II	CDCs	Intracoronary	Est 132	12 mo	Currently ongoing	N/A
HOPE (NCT02485938)	Phase I/II	CDCs	Intracoronary	Est 34	12 mo	Currently ongoing	N/A
DYNAMIC (NCT02293603)	Phase I	CDCs	Intracoronary	Est 42	12 mo	Currently ongoing	N/A
CAREMI (NCT02439398)	Phase I/II	CDCs	Intracoronary	Est 55	1, 6 & 12 mo	Currently ongoing	N/A
ESCORT (NCT02057900)	Phase I	ESC-derived ISL1^+^/ CD15^+^	Epicardial patch	N/A	N/A	Currently recruiting	N/A

For ongoing and currently recruiting trials with no published results, the NCT (national clinical trial) identifier has been indicated as referenced by http://ClinicalTrials.gov; CPCs: cardiac progenitor cells, MSCs: mesenchymal stem cells, CDCs: cardiosphere-derived cells, CABG: coronary artery bypass graft, LVEF: left ventricular ejection fraction, mo: month, Est: estimated, N/A: not applicable, ↑: increase, ↓: decrease.

## References

[1] Caspi O., Huber I., Kehat I. (2007). Transplantation of human embryonic stem cell-derived cardiomyocytes improves myocardial performance in infarcted rat hearts. *Journal of the American College of Cardiology*.

[2] Qian L., Huang Y., Spencer C. I. (2012). *In vivo* reprogramming of murine cardiac fibroblasts into induced cardiomyocytes. *Nature*.

[3] Sanganalmath S. K., Bolli R. (2013). Cell therapy for heart failure: a comprehensive overview of experimental and clinical studies, current challenges, and future directions. *Circulation Research*.

[4] Monteiro L. M., Vasques-Nóvoa F., Ferreira L., Pinto-do-Ó P., Nascimento D. S. (2017). Restoring heart function and electrical integrity: closing the circuit. *npj Regenerative Medicine*.

[5] Mauretti A., Spaans S., Bax N. A. M., Sahlgren C., Bouten C. V. C. (2017). Cardiac progenitor cells and the interplay with their microenvironment. *Stem Cells International*.

[6] Sahara M., Santoro F., Chien K. R. (2015). Programming and reprogramming a human heart cell. *The EMBO Journal*.

[7] Tzahor E., Poss K. D. (2017). Cardiac regeneration strategies: staying young at heart. *Science*.

[8] Moretti A., Caron L., Nakano A. (2006). Multipotent embryonic *isl1*^+^ progenitor cells lead to cardiac, smooth muscle, and endothelial cell diversification. *Cell*.

[9] Wu S. M., Fujiwara Y., Cibulsky S. M. (2006). Developmental origin of a bipotential myocardial and smooth muscle cell precursor in the mammalian heart. *Cell*.

[10] Brade T., Pane L. S., Moretti A., Chien K. R., Laugwitz K. L. (2013). Embryonic heart progenitors and cardiogenesis. *Cold Spring Harbor Perspectives in Medicine*.

[11] Liang X., Wang G., Lin L. (2013). HCN4 dynamically marks the first heart field and conduction system precursors. *Circulation Research*.

[12] Später D., Abramczuk M. K., Buac K. (2013). A HCN4+ cardiomyogenic progenitor derived from the first heart field and human pluripotent stem cells. *Nature Cell Biology*.

[13] Cai C. L., Liang X., Shi Y. (2003). Isl1 identifies a cardiac progenitor population that proliferates prior to differentiation and contributes a majority of cells to the heart. *Developmental Cell*.

[14] Bu L., Jiang X., Martin-Puig S. (2009). Human ISL1 heart progenitors generate diverse multipotent cardiovascular cell lineages. *Nature*.

[15] Laugwitz K. L., Moretti A., Lam J. (2005). Postnatal isl1^+^ cardioblasts enter fully differentiated cardiomyocyte lineages. *Nature*.

[16] Noseda M., Peterkin T., Simoes F. C., Patient R., Schneider M. D. (2011). Cardiopoietic factors: extracellular signals for cardiac lineage commitment. *Circulation Research*.

[17] Cai C. L., Martin J. C., Sun Y. (2008). A myocardial lineage derives from *Tbx18* epicardial cells. *Nature*.

[18] Zhou B., Ma Q., Rajagopal S. (2008). Epicardial progenitors contribute to the cardiomyocyte lineage in the developing heart. *Nature*.

[19] Katz T. C., Singh M. K., Degenhardt K. (2012). Distinct compartments of the proepicardial organ give rise to coronary vascular endothelial cells. *Developmental Cell*.

[20] Hutson M. R., Kirby M. L. (2007). Model systems for the study of heart development and disease: cardiac neural crest and conotruncal malformations. *Seminars in Cell & Developmental Biology*.

[21] Hildreth V., Webb S., Bradshaw L., Brown N. A., Anderson R. H., Henderson D. J. (2008). Cells migrating from the neural crest contribute to the innervation of the venous pole of the heart. *Journal of Anatomy*.

[22] Beltrami A. P., Barlucchi L., Torella D. (2003). Adult cardiac stem cells are multipotent and support myocardial regeneration. *Cell*.

[23] Chong J. J. H., Forte E., Harvey R. P. (2014). Developmental origins and lineage descendants of endogenous adult cardiac progenitor cells. *Stem Cell Research*.

[24] Bearzi C., Rota M., Hosoda T. (2007). Human cardiac stem cells. *Proceedings of the National Academy of Sciences of the United States of America*.

[25] Ellison G. M., Vicinanza C., Smith A. J. (2013). Adult c-kit^pos^ cardiac stem cells are necessary and sufficient for functional cardiac regeneration and repair. *Cell*.

[26] van Berlo J. H., Kanisicak O., Maillet M. (2014). c-kit^+^ cells minimally contribute cardiomyocytes to the heart. *Nature*.

[27] Smith A. J., Lewis F. C., Aquila I. (2014). Isolation and characterization of resident endogenous c-kit^+^ cardiac stem cells from the adult mouse and rat heart. *Nature Protocols*.

[28] Fazel S., Cimini M., Chen L. (2006). Cardioprotective c-kit^+^ cells are from the bone marrow and regulate the myocardial balance of angiogenic cytokines. *The Journal of Clinical Investigation*.

[29] Vicinanza C., Aquila I., Scalise M. (2017). Adult cardiac stem cells are multipotent and robustly myogenic: c-kit expression is necessary but not sufficient for their identification. *Cell Death & Differentiation*.

[30] Hong K. U., Guo Y., Li Q. H. (2014). c-kit+ cardiac stem cells alleviate post-myocardial infarction left ventricular dysfunction despite poor engraftment and negligible retention in the recipient heart. *PLoS One*.

[31] Torán J. L., Aguilar S., López J. A. (2017). CXCL6 is an important paracrine factor in the pro-angiogenic human cardiac progenitor-like cell secretome. *Scientific Reports*.

[32] Chong J. J. H., Reinecke H., Iwata M., Torok-Storb B., Stempien-Otero A., Murry C. E. (2013). Progenitor cells identified by PDGFR-alpha expression in the developing and diseased human heart. *Stem Cells and Development*.

[33] Kattman S. J., Huber T. L., Keller G. M. (2006). Multipotent flk-1^+^ cardiovascular progenitor cells give rise to the cardiomyocyte, endothelial, and vascular smooth muscle lineages. *Developmental Cell*.

[34] Kattman S. J., Witty A. D., Gagliardi M. (2011). Stage-specific optimization of activin/nodal and BMP signaling promotes cardiac differentiation of mouse and human pluripotent stem cell lines. *Cell Stem Cell*.

[35] Yang L., Soonpaa M. H., Adler E. D. (2008). Human cardiovascular progenitor cells develop from a KDR^+^ embryonic-stem-cell-derived population. *Nature*.

[36] Matsuura K., Nagai T., Nishigaki N. (2004). Adult cardiac Sca-1-positive cells differentiate into beating cardiomyocytes. *The Journal of Biological Chemistry*.

[37] Oh H., Bradfute S. B., Gallardo T. D. (2003). Cardiac progenitor cells from adult myocardium: homing, differentiation, and fusion after infarction. *Proceedings of the National Academy of Sciences of the United States of America*.

[38] Messina E., De Angelis L., Frati G. (2004). Isolation and expansion of adult cardiac stem cells from human and murine heart. *Circulation Research*.

[39] Martin C. M., Meeson A. P., Robertson S. M. (2004). Persistent expression of the ATP-binding cassette transporter, Abcg2, identifies cardiac SP cells in the developing and adult heart. *Developmental Biology*.

[40] DeLaughter D. M., Bick A. G., Wakimoto H. (2016). Single-cell resolution of temporal gene expression during heart development. *Developmental Cell*.

[41] Jain R., Li D., Gupta M. (2015). Integration of Bmp and Wnt signaling by Hopx specifies commitment of cardiomyoblasts. *Science*.

[42] Bardot E., Calderon D., Santoriello F. (2017). Foxa2 identifies a cardiac progenitor population with ventricular differentiation potential. *Nature Communications*.

[43] Ishida H., Saba R., Kokkinopoulos I. (2016). GFRA2 identifies cardiac progenitors and mediates cardiomyocyte differentiation in a RET-independent signaling pathway. *Cell Reports*.

[44] Birket M. J., Ribeiro M. C., Verkerk A. O. (2015). Expansion and patterning of cardiovascular progenitors derived from human pluripotent stem cells. *Nature Biotechnology*.

[45] Lalit P. A., Salick M. R., Nelson D. O. (2016). Lineage reprogramming of fibroblasts into proliferative induced cardiac progenitor cells by defined factors. *Cell Stem Cell*.

[46] Witman N., Sahara M. (2016). Expansion of cardiac progenitors from reprogrammed fibroblasts as potential novel cardiovascular therapy. *Stem Cell Investigation*.

[47] Zhang Y., Cao N., Huang Y. (2016). Expandable cardiovascular progenitor cells reprogrammed from fibroblasts. *Cell Stem Cell*.

[48] Fernandes S., Chong J. J. H., Paige S. L. (2015). Comparison of human embryonic stem cell-derived cardiomyocytes, cardiovascular progenitors, and bone marrow mononuclear cells for cardiac repair. *Stem Cell Reports*.

[49] Khan A. R., Farid T. A., Pathan A. (2016). Impact of cell therapy on myocardial perfusion and cardiovascular outcomes in patients with angina refractory to medical therapy: a systematic review and meta-analysis. *Circulation Research*.

[50] Hao M., Wang R., Wang W. (2017). Cell therapies in cardiomyopathy: current status of clinical trials. *Analytical Cellular Pathology*.

[51] Bolli R., Chugh A. R., D'Amario D. (2011). Cardiac stem cells in patients with ischaemic cardiomyopathy (SCIPIO): initial results of a randomised phase 1 trial. *The Lancet*.

[52] Nowbar A. N., Mielewczik M., Karavassilis M. (2014). Discrepancies in autologous bone marrow stem cell trials and enhancement of ejection fraction (DAMASCENE): weighted regression and meta-analysis. *BMJ*.

[53] Hare J. M., Fishman J. E., Gerstenblith G. (2012). Comparison of allogeneic vs autologous bone marrow–derived mesenchymal stem cells delivered by transendocardial injection in patients with ischemic cardiomyopathy: the POSEIDON randomized trial. *Journal of the American Medical Association*.

[54] Karantalis V., DiFede D. L., Gerstenblith G. (2014). Autologous mesenchymal stem cells produce concordant improvements in regional function, tissue perfusion, and fibrotic burden when administered to patients undergoing coronary artery bypass grafting: the Prospective Randomized Study of Mesenchymal Stem Cell Therapy in Patients Undergoing Cardiac Surgery (PROMETHEUS) trial. *Circulation Research*.

[55] Chimenti I., Gaetani R., Barile L. (2012). Isolation and expansion of adult cardiac stem/progenitor cells in the form of cardiospheres from human cardiac biopsies and murine hearts. *Methods in Molecular Biology*.

[56] Malliaras K., Makkar R. R., Smith R. R. (2014). Intracoronary cardiosphere-derived cells after myocardial infarction: evidence of therapeutic regeneration in the final 1-year results of the CADUCEUS trial (CArdiosphere-Derived aUtologous stem CElls to reverse ventricUlar dySfunction). *Journal of the American College of Cardiology*.

[57] Yacoub M. H., Terrovitis J. (2013). CADUCEUS, SCIPIO, ALCADIA: cell therapy trials using cardiac-derived cells for patients with post myocardial infarction LV dysfunction, still evolving. *Global Cardiology Science & Practice*.

[58] Burridge P. W., Matsa E., Shukla P. (2014). Chemically defined generation of human cardiomyocytes. *Nature Methods*.

[59] Murry C. E., Keller G. (2008). Differentiation of embryonic stem cells to clinically relevant populations: lessons from embryonic development. *Cell*.

[60] Xi J., Khalil M., Shishechian N. (2010). Comparison of contractile behavior of native murine ventricular tissue and cardiomyocytes derived from embryonic or induced pluripotent stem cells. *The FASEB Journal*.

[61] Chong J. J. H., Yang X., Don C. W. (2014). Human embryonic-stem-cell-derived cardiomyocytes regenerate non-human primate hearts. *Nature*.

[62] Shiba Y., Fernandes S., Zhu W. Z. (2012). Human ES-cell-derived cardiomyocytes electrically couple and suppress arrhythmias in injured hearts. *Nature*.

[63] Shiba Y., Filice D., Fernandes S. (2014). Electrical integration of human embryonic stem cell-derived cardiomyocytes in a guinea pig chronic infarct model. *Journal of Cardiovascular Pharmacology and Therapeutics*.

[64] Tang X. L., Rokosh G., Sanganalmath S. K. (2010). Intracoronary administration of cardiac progenitor cells alleviates left ventricular dysfunction in rats with a 30-day-old infarction. *Circulation*.

[65] Bolli R., Tang X. L., Sanganalmath S. K. (2013). Intracoronary delivery of autologous cardiac stem cells improves cardiac function in a porcine model of chronic ischemic cardiomyopathy. *Circulation*.

[66] Cimetta E., Godier-Furnemont A., Vunjak-Novakovic G. (2013). Bioengineering heart tissue for *in vitro* testing. *Current Opinion in Biotechnology*.

[67] Hirt M. N., Hansen A., Eschenhagen T. (2014). Cardiac tissue engineering: state of the art. *Circulation Research*.

[68] Tachibana A., Santoso M. R., Mahmoudi M. (2017). Paracrine effects of the pluripotent stem cell-derived cardiac myocytes salvage the injured myocardium. *Circulation Research*.

[69] Ieda M., Fu J. D., Delgado-Olguin P. (2010). Direct reprogramming of fibroblasts into functional cardiomyocytes by defined factors. *Cell*.

[70] Song K., Nam Y. J., Luo X. (2012). Heart repair by reprogramming non-myocytes with cardiac transcription factors. *Nature*.

[71] Yamakawa H., Ieda M. (2015). Strategies for heart regeneration: approaches ranging from induced pluripotent stem cells to direct cardiac reprogramming. *International Heart Journal*.

